# Development and Characterization of Lime-Based Mortars Modified with Graphene Nanoplatelets

**DOI:** 10.3390/ma17205022

**Published:** 2024-10-14

**Authors:** Adam Pivák, Milena Pavlíková, Martina Záleská, Zbyšek Pavlík

**Affiliations:** Department of Materials Engineering and Chemistry, Faculty of Civil Engineering, Czech Technical University in Prague, Thákurova 7, 166 29 Prague, Czech Republic; adam.pivak@fsv.cvut.cz (A.P.); milena.pavlikova@fsv.cvut.cz (M.P.); martina.zaleska@fsv.cvut.cz (M.Z.)

**Keywords:** graphene nanoplatelets, hydrated lime, hydraulic lime, nanoadditive, structural characteristics, strength enhancement, drying rate

## Abstract

Materials for the conservation of cultural heritage must meet specific demands, such as high durability, service life, and compatibility with other materials used in the original building structures. Due to their low permeability to water and water vapor and their high rigidity, the use of Portland cement (PC) mortars, despite their high mechanical resistance and durability, does not represent an appropriate solution for the repair of historic masonry and structures. Their incompatibility with the original materials used in the past, often on a lime basis, is therefore a serious deficiency for their application. On the other hand, lime-based mortars, compared to PC-based materials, are more susceptible to mechanical stress, but they possess high porosity, a high water vapor transmission rate, and moderate liquid water transport. This study aims at the development of two types of lime-based mortars, calcium lime (CL) and hydraulic lime (HL). The modification of mortars was conducted with a carbon-based nanoadditive and graphene nanoplatelets (GNs) in three dosages: 0.1%, 0.3%, and 0.5% of the binder weight. The enhancement of CL mortars by GNs greatly increased mechanical strength and affected heat transport characteristics, while other characteristics such as porosity, water absorption, and drying rate remained almost similar. The application of GNs to HL not only enhanced the strength of mortars but also decreased their porosity, influenced pore size distribution, and other dependent characteristics. It can be concluded that the use of graphene nanoplatelets as an additive of lime-based composites can be considered a promising method to reinforce and functionalize these composite materials. The improved mechanical resistance while maintaining other properties may be favorable in view of the increasing requirements of building materials and may prolong the life span of building constructions.

## 1. Introduction

Given the historical evidence, the first mortars used were based on gypsum, mud, and lime. Due to the specific characteristics and service life, non-hydraulic and sub-hydraulic mortars mainly composed of calcined lime were used until the 19th century. Between the 19th and 20th centuries, traditional lime was replaced by materials based on hydraulic lime and Portland cement (PC) [1,2]. PC has since become the most widely used binder in construction today. According to the European Cement Association, global cement production was estimated at approximately 4.1 billion tons in 2022 [3]. However, despite good properties, such as high durability, mechanical strength, corrosion resistance, and availability, the use of PC-based materials is not always a good choice due to the specific requirements on construction properties, especially for the reconstruction of old or even heritage buildings. Compared to PC, lime-based materials have a significantly higher water-vapor permeability, which is generally preferable to eliminate the potential damage associated with moisture and salt action. The high permeability of the mortar ensures that water evaporates before spreading throughout the structure and prevents further damage [4]. In some cases, even the excellent mechanical strength and lower thermal expansion characteristics of PC-based mortars may be unnecessary or even undesirable, especially in the repair of historic building, as other original inbuilt construction materials have considerably lower strength and rigidity [5].

For these reasons, it is important to consider whether the use of PC mortars in repair and restoration is appropriate, as the incompatibility of old and new materials may lead to additional damages related to construction, hygrothermal, and other problems [4,5,6,7]. When repairing historic buildings, we must keep in mind not only the functional aspects of the materials used, but also the preservation of their historical value and authenticity. Therefore, authorities responsible for cultural heritage emphasize the use of compatible materials, preferably the same ones used in the original state of the building [8,9]. However, increasing demands on building structures are driving the research and design of modified materials that achieve higher durability with the least interference to other material properties. One possibility is the use of nanoscale materials that have attracted the attention of researchers in recent years.

The construction industry is now focusing on the potentially promising possibilities of incorporating nanomaterials (NMs) into building materials to improve their properties. Given the variety of NM types and incorporation methods available in terms of preparation or applicable volumes, the effect of nano-additives must be properly evaluated to achieve their optimal application in composites, considering the high price of NMs. There are several studies aimed at improving the mechanical properties of building composites, mostly concrete, by incorporating different types of carbon-based nano-admixtures.

In a study conducted by Jeevanagoudar et al. [10], multi-walled carbon nanotubes (MWCNTs) were added to a mortar mixture after being dispersed through a magnetic stirrer followed by sonication, without using any chemical dispersants. A volume of 0.4 wt.% (NM to binder ratio) of MWCNTs improved the compressive strength by 17%, while the half amount of NM, 0.2 wt.%, led only to a 7% improvement. On the other hand, increasing the dose to more than 0.4% by weight resulted in reduced strength. In contrast, X. Song et al. [11] reported a maximum increase in strength of only 3.5% for 0.25 wt.% of MWCNTs, although a dispersant, Polyvinyl Pyrrolidone (PVP), was employed. Improvement can be achieved even in low volumes of applied NMs, as highlighted in a study by Dalla et al. [12]. Authors found that dispersing nanoparticles by ultrasonication, aided by a polycarboxylate-based superplasticizer, led to a strength increase of 26% at a dose of 1.0 wt.% of graphene nanoplatelets (GNs). Similarly, Chen et al. [13] used a comparable dispersing method, achieving an even higher increase in compressive strength to more than 55 MPa, over 28%, with just 0.04 wt.% of GNs. However, as the concentration of GNs was further increased, the compressive strength decreased to 50 MPa when 1.0 wt.% of GNs was applied. Lauermannová et al. [14] studied the enhancement effect of the combination of GNs and MWCNTs on the alternative PC binder, magnesium oxychloride cement (MOC). NMs were dispersed with the support of tannic acid, which led to an approximate 30% increase in strength. Azunna et al. [15] reported on the improvement of the mechanical strength of the rubberized geopolymer concrete by GNs. The addition of GNPs by weight of the binder at 0.1%, 0.2%, 0.3%, and 0.4% raised the compressive strength by 6%, 12%, 18%, and 14%, respectively, with respect to the control specimen, with 0.3% producing the best result.

Recently, there has been considerable interest in the use of various forms of graphene derivatives to produce high-strength concrete. Among them, the superstructure of graphene quantum dots (GQDs) was studied as an innovative additive to improve the properties of PC composites [16]. Graphene quantum dots (GQDs) are CDs with a 2D structure of a single or few graphene layers, which possess enhanced mechanical strength, chemical stability, durability, corrosion resistance, low cost, and non-toxicity [17]. Win et al. [18] carried out research on the chemo-physical effect of superstructure GQDs on the microstructure and strength of PC mortars. The composite containing 1.2% supra-GQDs had higher compressive and flexural strengths than the control by 40% and 108% respectively. In addition, the use of supra-GQDs resulted in microstructural bridging and refinement of the pore structure.

Studies that focused on the applications of NMs in other types of building materials, such as lime-based mortars, have only occasionally been reported. A.-E. Dimou et al. [19] investigated the enhancement of lime–cement pastes using functionalized MWCNTs. Although a higher dosage of MWCNTs was used, this modification proved largely ineffective, resulting in only a 2% increase in mechanical resistance. In another study, Dimou et al. [20] explored the effects of adding 0.15 wt.% of functionalized MWCNTs and reduced graphene oxide (rGO) to natural hydraulic lime pastes (NHL). The MWCNTs produced similar results to the previous study, with only a 3% increase in strength. However, the use of rGO led to a substantially greater improvement, reaching a 20% increase in mechanical strength. However, in a study conducted by Faria et al. [21], the application of GO in NHL mortar increased the strength by only 12.5% with a dosage of 0.1 wt.%.

As can be seen, the results vary between individual studies with different types of NMs applied. Thus, every variant must be studied properly. Furthermore, the performance of composite materials is affected not only by the properties of the incorporated NMs themselves but also by their distribution within the bulk of the material, a factor that can be challenging to control. Nanoparticles often tend to aggregate due to weak van der Waals forces that cause the non-uniform distribution and formation of clusters, both in the dry state and in solution [22,23,24]. When NMs are incorrectly dispersed within the material, the formed clusters can act as weak points, leading to reduced strength. To achieve an effective distribution of NMs, these forces must be surpassed to ensure proper dispersion and incorporation of nanoadditives into the material.

This study focuses on the design and evaluation of lime-based mortars enriched by carbon-based nanoadditives—graphene nanoplatelets. The aim is to find and evaluate the optimal method of GNs in mixing and homogenization, the identification of the effective volume of applied nanoadditives, and the experimental assessment of the effect of nano-modification of mortars on their structural characteristics, mechanical strength, heat transport and storage parameters, water absorption and ingress, and drying ability. The nano-modified mortars were researched to find prospective materials for the repair of historic building stock and other specific construction applications. Thus, the research presented attempts to fill the gap in the modification of the properties and performance of lime-based mortars using carbon-based nanoadditives, taking into account the problems related to their homogeneous distribution in the fresh mixture and their effective dosage in terms of the final properties of the hardened mortars. As heritage authorities are not opposed to the functionalization of repair materials by nanoadditives, the incorporation of graphene-based nanoadditives can contribute to the design and development of durable and functional mortars for the repair of heritage buildings without the use of often “banned” PC.

## 2. Materials and Methods

In this experimental study, lime mortars based on lime hydrate (CL) and hydraulic lime (HL) modified with carbon-based nanoadditive were investigated. First, the characterization of both binders and filler was carried out. This included an assessment of their basic physical parameters, evaluation of the chemical composition, and particle size distribution. In the case of a chosen nanoadditive, the effectiveness of several dispersion techniques for its uniform distribution in water was investigated. The data obtained served as basic initial information for the preparation of mortar mixes.

### 2.1. Materials and Sample Preparation

As binders, lime hydrate CL 90-S (Čertovy schody a.s., Lhoist group, Tmaň, Czech Republic) and hydraulic lime HL 5 (Baumit GmbH, Waldegg, Austria) were used. Silica sand (Filtrační písky Ltd., Chlum u Doks, Czech Republic) consisting of a blend of three fractions referred to as PG1, PG2, and PG3 with grain size 0–0.5 mm, 0.5–1.0 mm, and 1.0–2.0 mm, respectively, was used as aggregate. All fractions were mixed in a 1: 1: 1 ratio to form a filler with a total fraction of 0–2 mm. The composition of the mortars studied is presented in Table 1. The binder to filler mass ratio was set at 1:3, similar to the testing cement strength. The amount of batch water was adjusted to achieve a similar workability of all mortar mixtures. The flow table test consistency (spread dimeter) was set to be 165 ± 5 mm, which is a common value for manually applied repair mortars.

The graphene nanoplatelets C-750 (Sigma Aldrich, St. Luis, MO, USA) were the incorporated carbon-based nanoadditive. The GNs had a particle size of ~2 µm and a high specific surface area (750 m^2^·g^−1^), which is, according to Lauermannová et al. [25], beneficial for bonding the NMs to the matrix of composite materials. GNs were applied at a dose of 0.1%, 0.3%, and 0.5% of the weight of the used binder (samples labeled GN0.1, GN0.3, and GN0.5). The content of GNs in the composition of the mortars was chosen, taking into reference our previous studies on the nanomodification of MOC composites, and the analysis of the relevant literature sources [14,15].

Both types of mortars were prepared using a similar preparation procedure. In the case of the nano-modified mixtures, the first step was the dispersion of graphene nanoplatelets in a part of batch water. The choice of a nanoadditive dispersion technique is discussed in Section 3.2. The dispersion of NM itself was carried out for 10 min. When the dispersion stopped, the nanosuspension was mixed with the binder and the rest of the batch water using a planetary mixer (ELE International, Milton Keynes, UK), followed by the addition of the aggregate, according to the standard EN 459-2 [26]. The prepared mixtures were placed in plastic prismatic molds (40 mm × 40 mm × 160 mm), demolded after 48 h, and then stored in a climatic chamber at a temperature *T* = (23 ± 2) °C. The relative humidity (*RH*) varied with respect to the binder type used. The CL mortars were cured at a relative humidity of (60 ± 5) % to support their carbonation and hardening while the HL mortars were cured at a higher *RH* = (90 ± 5) % to ensure the hydration process of the samples and to avoid the formation of drying cracks. The total curing time under these conditions was 28 days. In addition, the second set of the samples was then left under laboratory conditions at *T* = (23 ± 2) °C and *RH* = (40 ± 5) % until tested at the age of 365 days.

### 2.2. Evaluation of Nanoadditive Dispersion Technique

As already stated above, the resulting properties of the composite material modified with nanoadditives were influenced not only by the characteristics of the NMs themselves, but also by their uniform distribution in the composite. Inadequate homogenization of the nanomaterial in the composite matrix results in the formation of clusters and weak spots that are held by weak attractive van der Waals forces, which results in the insufficient utilization of the extraordinary properties of the NMs and, in some cases, can lead to the deterioration of the final properties. For this reason, it was necessary to determine the most efficient method for the preparation of graphene nanoplatelets dispersion.

Based on studies [27,28,29,30,31,32,33,34,35,36], four techniques to disperse graphene nanoplatelets were studied. Among them, the stirring method using a magnetic stirrer (IKA-Werke GmbH & Co. KG, Staufen, Germany), sonication with an ultrasonic bath Elmasonic Easy 60 H (Elma Schmidbauer GmbH, Singen, Germany), mixing with a high-shear mixer UltraTurrax T-18 (IKA) and homogenization using a Turbula T2F shaker-mixer (Willy A Bachofen AG, Muttenz, Switzerland) were analyzed.

The level of nanoadditive dispersion achieved by each technique was evaluated based on a visible light absorption, which was measured using a UV/VIS instrument GENESYS 30 Visible Spectrophotometer (Thermo Scientific^TM^, Waltham, MA, USA). The assessment was based on the assumption that when nanoparticles are dispersed in a liquid, the surface of these nanoparticles absorbs UV radiation and visible light passing through the liquid. As the level of dispersion of the nanomaterial in suspension increased, a higher absorbance was measured at each wavelength.

### 2.3. Characterization of Binders and Aggregate

The loose bulk density of all raw materials was determined by the gravimetric method. The specific density was determined using a Pycnomatic ATC helium pycnometer (Thermo Scientific^TM^, Waltham, MA, USA). The specific surface area was measured by an air permeability test using a Blaine UTCM-0280 automated apparatus (UTEST, Ankara, Turkey).

The chemical composition of the binders and aggregate used was measured using an ARL Quant’X EDXRF spectrophotometer (Thermo Scientific^TM^, Waltham, MA, USA).

Two methods were used to determine the particle size distribution. The particle size of the binders was analyzed by the laser diffraction method using an Analyssete 22 Micro Tec plus (FRITSCH GmbH, Weimar, Germany). The particle size of silica sand was larger; thus, the grain size was measured by a sieving method according to EN 1015-1 [37].

### 2.4. Methods of Testing Fresh and Hardened Mortars

The workability of fresh mortars was characterized in a flow table test carried out in accordance with EN 1015-3 [38].

Hardened mortars subjected to a 28-day curing regime were used to determine the basic physical properties and mechanical parameters. In addition, the testing of the 365-day mortars included the evaluation of the heat transfer, storage capacity, and the assessment of the hygric properties. A summary of the experimental tests performed on the hardened mortars, the corresponding test methods, and the expanded combined uncertainties of the material parameters assessment is given in Table 2.

The basic structural characteristics of hardened mortars, namely bulk density, specific density, and porosity were tested on 5 specimens dried in a vacuum chamber at 60 °C until their steady state mass was achieved. The bulk density was measured according to the standard EN 1015-10 [39] using the gravimetric method. The specific density was determined by helium pycnometry (see Section 2.3). The total open porosity was calculated from the resulting bulk density and specific density obtained in previous tests. In the case of samples cured for 365 days, the porous structure was subjected to detailed characterization with mercury intrusion porosimetry (MIP) using a set of two measurement modules, Pascal 140 and 440 (Thermo Scientific^TM^).

The mechanical resistance of the mortars investigated was characterized by the flexural strength of prism-shaped specimens (40 mm × 40 mm × 160 mm) and the compressive strength measured on fragments from the flexural strength test with a loading area of 40 × 40 mm^2^. Both mechanical tests were carried out using a mechanical press FP 100 (Heckert, Rauenstein, Germany) in compliance with the technical standard EN 1015-11 [40].

Young’s modulus testing was performed on vacuum chamber dried specimens using a Vikasonic, ultrasonic testing device (Schleibinger Geräte GmbH, Buchbach, Germany). The Young’s modulus was calculated based on the detection of the ultrasonic wave velocity as specified in EN 12504-4 [41].

Thermal conductivity and volumetric heat capacity of mortars were the tested thermal parameters. They were obtained on transient plane source technique principles using a TPS 1500 hot disk apparatus (Hot Disk AB, Göteborg, Sweden). The measurement was carried out on dry specimens at a temperature of 23 ± 2 °C. Hygric parameters, the water absorption coefficient, the 24 h water absorption, and the apparent moisture diffusivity were obtained in a capillary water uptake experiment [42,43].

The drying rate and the drying index were measured according to the EN 16322 standard [44]. The 40 mm cubes cured for 365 days were immersed in water until fully saturated. The samples were then covered with a permeable vapor film on all sides (except one that served as an evaporation surface) and were subjected to drying in a controlled environment at *T* = (23 ± 2) °C and *RH* = (50 ± 5) % for 10 days. The mass of the specimens was periodically recorded to calculate the drying rate.

## 3. Results

### 3.1. Characterization of Raw Materials

The chemical composition of both binders, presented in Table 3, shows a high concentration of the main component, CaO, which is typical for lime hydrate. Hydraulic lime also contains a higher amount of hydraulic oxides (SiO_2_, Al_2_O_3_, Fe_2_O_3_). In the case of silica sand, quartz was identified as the main component. In addition, the presence of Al_2_O_3_ was also recorded.

The basic physical characteristics of lime hydrate (CL 90-S), hydraulic lime (HL 5), and silica sand used are summarized in Table 4. The tested lime hydrate showed significantly higher fineness compared to that of hydraulic lime. Due to the larger fraction of aggregate used, its Blaine specific surface area was not measured.

**Figure 1:**
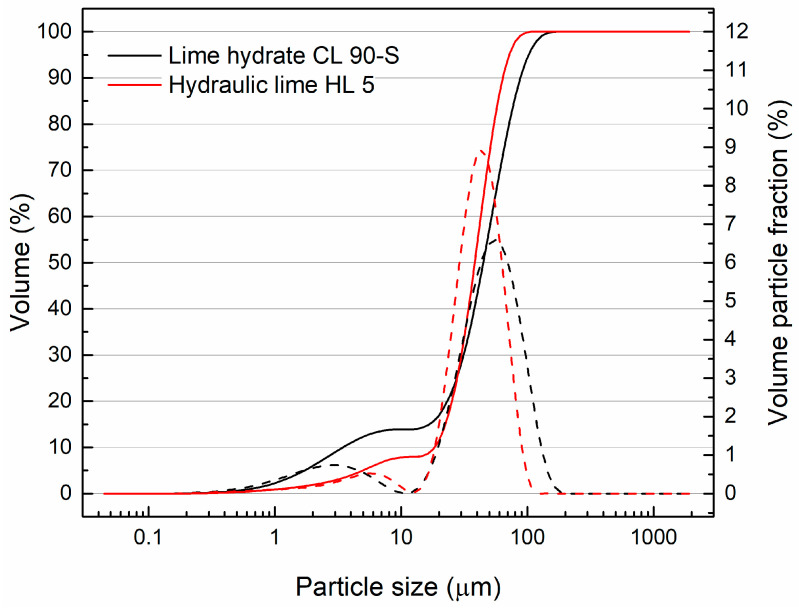
Particle size distribution of used binders.

**Figure 2:**
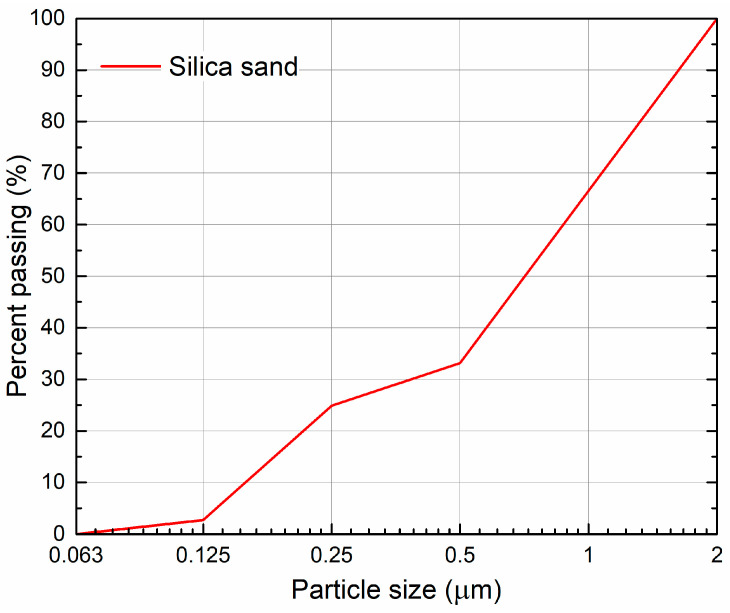
Grading curve of silica aggregate.

Compared to hydraulic lime, the particle size distribution of lime hydrate (Figure 1) shows a larger volume of particles in the 0.5~5.5 μm size range. In contrast, hydraulic lime contains a higher proportion of particles in the 18.0~65.4 μm size range, which corresponds well with its lower fineness as evaluated by the Blaine permeability test. The grain size curve of aggregated used is graphed in Figure 2.

### 3.2. Dispersion Technique

**Figure 3:**
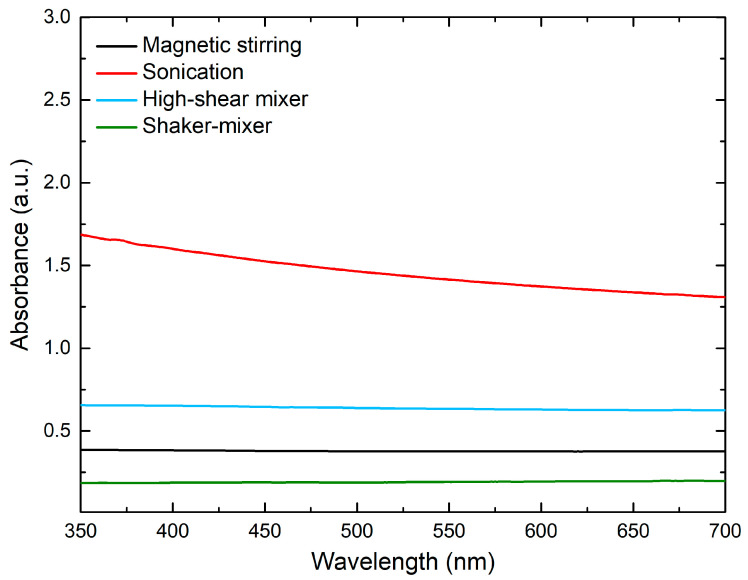
UV/VIS absorption spectra of GNs.

The effectiveness of techniques tested for the dispersion of GNs in tap water is illustrated in Figure 3. The results show that the dispersion of the nanoadditive realized by sonication in an ultrasonic bath achieved the highest absorption at all wavelengths, which can be attributed to an increased surface area of the GNs in suspension exposed to UV light. In contrast, the other methods used were several times less effective.

**Figure 4:**
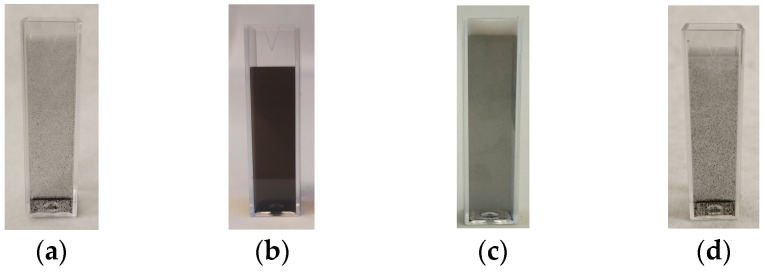
Sample cuvettes for dispersion test: (**a**) magnetic stirring, (**b**) sonication, (**c**) high-shear mixing, and (**d**) shaker-mixer.

The dispersion of GNs for all tested application methods is shown in Figure 4. In agreement with the results of UV/VIS spectroscopy, the sonication resulted in the most homogeneous dispersion of graphene nanoplatelets in water. The worst dispersion of GNs was achieved by shaker mixing. Considering the results of UV/VIS spectroscopy and visual inspection, the ultrasonic bath was used to prepare the nanoadditive dispersion for mortar mixing in this study.

It is worth noting that the GNs were dispersed in H_2_O only. Other studies [14,45,46,47,48,49,50] explored the possibilities of using chemical agents that support dispersion and its long-term stability. However, some commonly used chemicals, such as surfactants, also have the ability to stabilize air bubbles induced during mixing, which can negatively affect the final properties of the composite material.

### 3.3. Properties of 28-Day and 365-Day Hardened Mortars

The basic structural properties of mortars cured for 28 and 365 days are presented in Table 5. The GNs affected the investigated physical parameters of CL mortars only slightly. The differences in the parameters measured were typically in the range of measurement uncertainty. Compared to 28-day samples, the 1-year samples showed a small increase in bulk density and matrix density, followed by a slight reduction in porosity due to the prolonged carbonation of the lime mortars.

In the case of HL mortars, the addition of GNs resulted in an increase in the bulk density of more than 9% with a 0.1% and 0.3% nanoadditive dosage and 7.5% when 0.5 wt.% of GNs was applied. The resulting values of open porosity decreased approximately by 6% (absolute value) as compared to the control hydraulic lime mortar HL-R.

**Figure 5:**
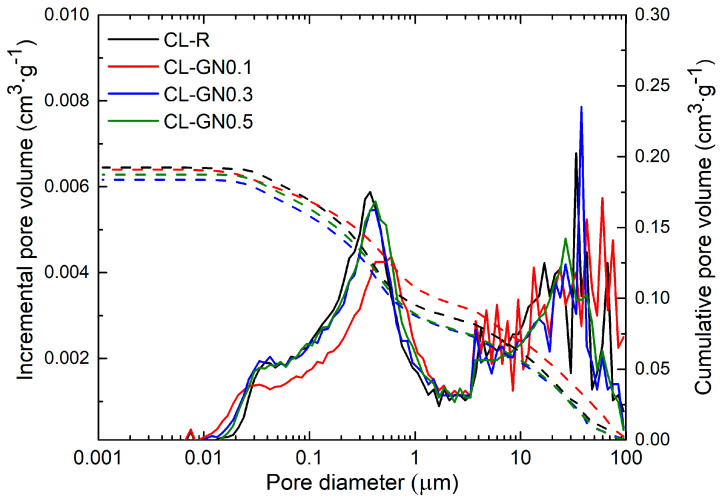
Pore size distribution of hardened CL mortars.

The results of the mercury intrusion porosimetry analysis of the microstructure of CL mortars (Table 6) also show that the effect of the GNs on pore volume and pore size was not significant. The control sample reached a total pore volume of nearly 0.2 cm^3^·g^−1^ and a mean pore diameter slightly greater than 0.2 µm. For CL mortars modified by the incorporation of GNs, only a small decrease in both parameters occurred. According to the distribution of pore size shown in Figure 5, the highest pore volumes were recorded for macro pores at around 12 µm in diameter and capillary pores in the range from 0.03 µm to 1 µm, which may lead to faster water absorption induced by capillary action. Although the average pore diameter was reduced by GNs admixing, the volume of pores with a diameter > 0.4 µm, i.e., those enabling water transport, was increased as compared to the reference material CL-R.

**Figure 6:**
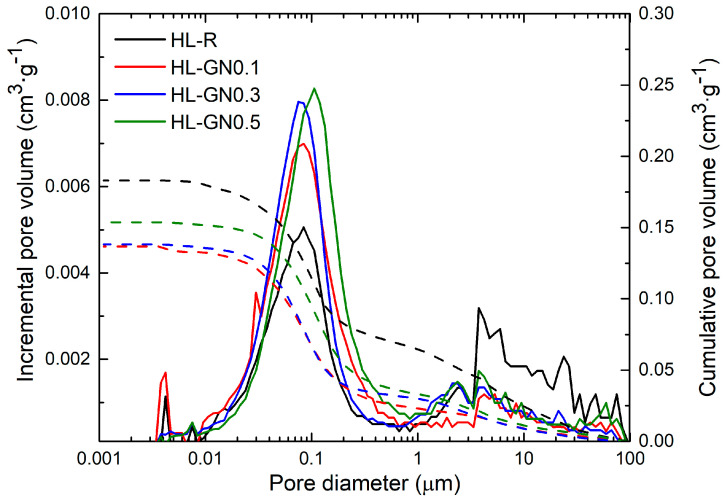
Pore size distribution of hardened HL mortars.

The pore size distribution is shown in Figure 6. The dotted lines represent cumulative pore size distribution curves, the solid line the incremental curves. The porosity distribution of the HL mortars revealed the presence of smaller pores compared to the CL mortars. The main volume of the capillary pores had a diameter in the range of 0.01 µm to 0.3 µm. The average pore diameter of the modified mortars was around 0.04 to 0.08 µm. It is also necessary to consider that, compared to mortars modified with GNs, the control mortar, HL-R, had higher total open porosity and less capillary pores in the same range and a higher volume of macro pores between 3 µm and 30 µm, which can further reduce the rate of water absorption.

The resistance of hardened CL and HL mortars to withstand mechanical stress characterized by flexural strength, compressive strength, and dynamic modulus of elasticity is presented in Table 7. GN modification of CL mortars resulted in an increase in all mechanical parameters investigated. The highest flexural strength was obtained for mortar CL-GN0.1. Mortars with a 0.3% and 0.5% dosage of nanoadditives reached a higher flexural strength compared to the control sample; however, it was not as high as CL-GN0.1. Similarly, the flexural strength of HL mortars increased as compared to the reference but not such a high extent as recorded for CL mortars.

For both types of mortars studied, the highest compressive strength values were obtained with the addition of GNs in a dosage of 0.3 wt.%. For 28-day samples, the strength gain was approx. 49.1% for CL-GN0.3 and approx. 50.6% for mortar HL-GN0.3. While in the case of CL samples, the increase can be attributed to the nanoadditive used, i.e., its excellent mechanical parameters and the strength increase in HL mortars can be explained by the combination of two factors: the effect of the nano-reinforcement and the reduction in the total open porosity. Further increasing the content of GNs in the composition of mortars to 0.5 wt.% reduced their final strength due to unevenly distributed nanoplatelets in high doses and the subsequent formation of agglomerates, which affected the interfacial area of the composite matrix and weakened the overall performance of the mortars studied [51].

The nanomodification of mortars not only had a significant influence on the mechanical performance of the 28-day samples, but it was also confirmed for the samples that underwent a longer curing. In this case, the highest compressive strength was also recorded for mortars with a 0.3% dose of GNs, but the final strength increase compared to the control materials reached only 14.5% and 29.9% for CL and HL samples, respectively. It is thus evident that the applied nanoadditive competed with continuous carbonation and, in the case of HL samples, also with prolonged hydration and densification of hydrated phases in the improvement of compressive strength.

The use of GNs also resulted in the enhanced dynamic Young’s modulus. The improvement of this mechanical parameter was obviously more significant in the case of HL mortars, where the increase in this parameter varied in the range of 11.1–23.3% for 28-day samples and 19.6–20.1% for 365-day samples. Similarly, as in the evaluation of compressive strength, the gain in the dynamic modulus of elasticity was due to the extraordinary properties of GNs themselves combined with the solidified matrix of HL samples.

The thermal parameters of hardened mortars are shown in Table 8. The heat transfer and storage were significantly affected by the addition of GNs. Both thermal conductivity and volumetric heat capacity values increased with an increasing volume of the applied nanoadditive. For CL mortars, the increase was lower, as the samples with CNs and those of reference had nearly identical total porosity. The highest increase in the thermal conductivity and volumetric heat capacity, recorded for CL-GN0.5, was more than 9%. The increase in the thermal conductivity values was mainly due to the high thermal conductivity (~4000 W·m^−1^·K^−1^) of graphene [52].

In the case of HL mortars, the increase in both thermal parameters due to the addition of GNs was greater, about 26% and 28% in thermal conductivity and volumetric heat capacity, respectively. The changes in the thermal parameters were due to a combination of the effect of the graphene addition and a decrease in the total porosity of the graphene-enriched mortars compared to the control HL-R sample. The differences in both thermal parameters of the samples with different doses of GNs were only minimal.

**Figure 7:**
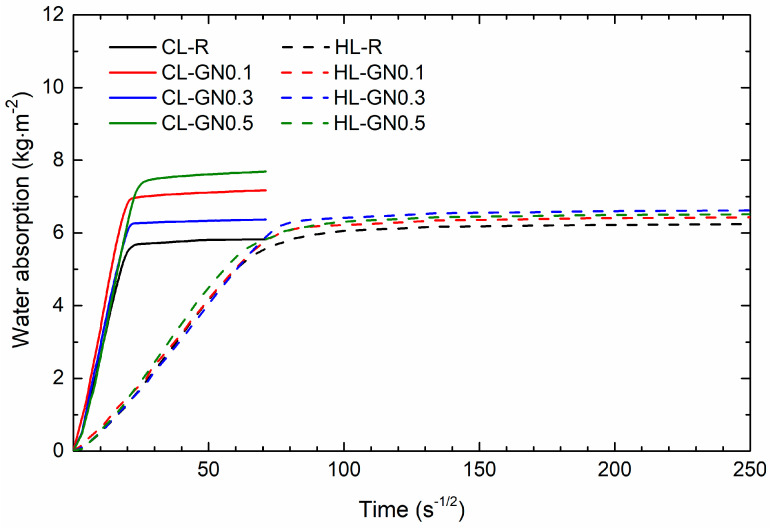
Capillary water absorption curves of 365-day cured mortars.

The results of the capillary water absorption test and the average water absorption curves of the mortars cured for 365 days are shown in Table 9 and Figure 7. The control lime mortar sample, CL-R, reached a water absorption coefficient of more than 0.33 kg·m^−2^·s^−1/2^, which is typical for lime mortars, as presented in other studies [53,54,55]. The absorption rate increased with the addition of nanomaterials as well as the total amount of capillary absorbed water after 24 h due to changes in pore volume and distribution. However, these changes were only slightly higher than the combined uncertainty of the test. In contrast, the reference HL mortar reached a water absorption rate approximately 10 times lower than CL-R. This low absorption rate was mainly because the HL-R mortar had a lower volume of capillary pores accessible for water and enabling its transport. Moreover, the hydraulic lime used was an industrially prepared blend of Portland cement and lime hydrate. As Portland cement mortars are known for a low ability to transport liquid water, the presence of PC in the prepared mortars resulted in decelerated water transport and a reduced water absorption rate. The modification of HL mortars with GNs slightly increased both measured hygric parameters, which corresponds well with the pore size distribution curves presented in Figure 6.

**Figure 8:**
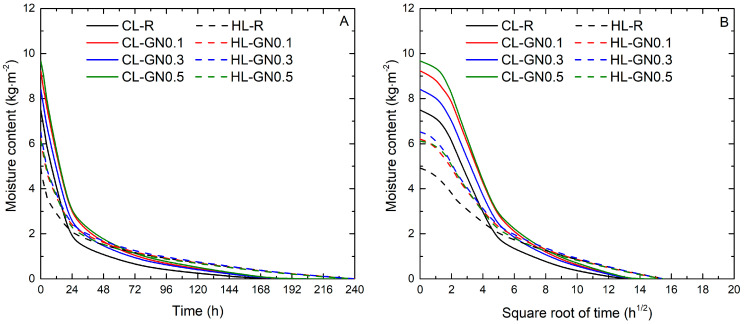
Evaporation curves of mortars cured for 365 days. (**A**) initial drying curves—phase I (**B**) drying curves—phase 2.

The curves representing the one-dimensional drying process are shown in Figure 8. The drying mechanism can be divided into two phases. The first phase represents the drying from the evaporation front on the fully saturated surface of the samples to the controlled environment. This phase, also called the constant drying rate period [56], is illustrated as a straight line of evaporation as a function of time (h) presented in Figure 8A. The drying rate of this phase (*D*_1_) was calculated as a slope of this linear part of the curve. The second phase, the non-linear part of the drying curve, consists of the water evaporation and moisture diffusion that occurred when the evaporation front moved from the sample surface into the material. For calculation, the drying was plotted as a function of the square root of time (h^1/2^), shown in Figure 8B, and the drying rate of the second phase (*D*_2_) was quantified as a slope of the linear part of this curve.

The drying rate of both evaporation phases and the drying index describing how slowly the drying occurs are shown in Table 10. Similar to the resulting capillary water absorption data, the differences between the CL-R and modified CL mortars were only small, specifically for the first phase drying rate and the overall drying index. In the case of the Phase 2 drying rate, the differences between the reference and GN modified samples were more distinct, pointing to the higher moisture diffusivity of CL-GN mortars. Nevertheless, the drying index of mortars with GNs was higher compared to the reference mortar, i.e., the drying was slightly prolonged. However, one must consider that the drying started from a higher initial moisture content.

Larger differences between the control and modified samples can be observed for the HL mortars. In comparison to reference mortar HL-R, the changes in porous structure after the application of nanoadditives resulted in a lower drying index DI, i.e., led to faster water evaporation and water vapor transport through the sample during the drying test, which is consistent with the examined water absorption characteristics, as was also shown in other studies [57,58]. Even in this case, the differences between samples with different doses of GNs were only marginal. Thus, the results were not mainly dependent on the volume of applied nanomaterials but strictly on their presence.

## 4. Conclusions

In this study, lime-based mortars modified with graphene nanoplatelets were preprepared and experimentally investigated. The influence of the dosage of the nanoadditive used on the structural, thermal, hygric, and drying characteristics of the hardened mortars was thoroughly analyzed.

Based on the results of the experiments carried out, the following findings were highlighted:

(1) Using a UV/VIS spectroscopy test, sonication in ultrasonic bath was identified as the best nanomaterial dispersion technique. In this way, the optimal application of graphene nanoplatelets and their uniform distribution in the fresh mortar mixture were achieved.
(2) Nano-modified hydrated lime (CL) mortars were only slightly affected in terms of bulk density, matrix density, porosity, and pore size distribution, while maintaining the same workability of fresh mixtures. However, by modifying hydraulic lime mortars (HL), the bulk density and porosity parameters were significantly changed. The bulk density was increased by the addition of GNs in mortar composition by about 7.5–9.0%. The porosity dropped by approx. 20%. Except for the decrease in the total pore volume, the HL-GN mortars yielded a significantly lower average pore diameter as compared to the reference material HL-R.
(3) In case of modified mortars, the changes in pore structure caused by the application of GNs had a significant influence on water absorption and drying parameters. The heat transport and storage parameters were influenced by the high thermal conductivity of graphene itself and in the case of HL mortars through the reduced porosity of mortars. Thus, increased thermal conductivity and volumetric heat capacity were observed for all modified mortars studied.
(4) The nano-modification of mortars had a positive influence on their flexural and compressive strength. Similarly, the dynamic Young’s modulus was enhanced by the addition of GNs. In particular, the high-strength improvement of CL mortars, which exhibited a compressive strength nearly 50% higher compared to the control sample, is a very promising finding for their intended use in the repair of historical masonry.
(5) In view of the mechanical tests results, the advantage of excellent graphene properties was taken even when the lowest dosage of a nanoadditive (0.1 w.% to the binder) was applied. Contrary to that, increasing the dose of GNs to 0.5 wt.% led to a decrease in mechanical strength, possibly due to the formation of agglomerates of nanoparticles. In this respect, it should also be noted that although the 0.1% dose of GNs did not bring the highest mechanical strength, the difference between GN0.1 and GN0.3 was relatively low for CL mortars (0.10 MPa) and HL (0.21 MPa) cured for 28 days, compared to the cost of GNs, which was, for the 0.3% dose, three times higher. Therefore, the use of nanoadditives in higher volumes does not seem to be economically effective.

In general, it can be concluded that the application of graphene nanoplatelets as an additive to lime-based mortars is a promising method to reinforce and functionalize these composite materials. The excellent and unique properties of GNs make it possible to improve mechanical resistance while maintaining other functional properties of lime-based mortars, thus preserving their compatibility with the original materials of historical buildings. This compatibility can be favorable in view of the current increasing demands on building materials and the prolongation of the life of building structures. The ability to improve the mechanical parameters of CL-based mortars, which usually suffer from low mechanical resistance and durability, can be effectively exploited in the design of repair mortars. Similarly, the enhanced drying index of HL mortars can be utilized in the drying of damp buildings and in the transport of absorbed water to the drying front. In view of the technical and compatibility criteria imposed on repair mortars, the applicability of nano-dopants to improve their properties must be assessed on a case-by-case basis by the heritage authorities. In addition, the financial aspects must also be considered when selecting the appropriate repair materials.

It should also be noted that there is a wide variety of nanomaterials in terms of dimensions, such as dots or nanotubes with a length greater than 50 nm, or a chemical basis. Moreover, from a financial point of view, less pure (industrial type) nanoadditives might be used. In any case, based on the nanoadditive type and properties, a specific approach for their mixing and incorporation into composites must be designed, and the final properties of the developed composite mortars will be affected differently, which requires further complex analyses and tests. In this context, durability issues must be addressed.

Future research will focus on the workability of the prepared mortars and their adhesion to the substrate, which is necessary for their traditional and craft application.

## Figures and Tables

**Table 1:** Composition of the mortars studied (in g).

Mortar Mix	Lime Hydrate	Hydraulic Lime	Water	Silica Sand	Graphene
CL-R	1500	-	1575	3 × 1500	-
CL-GN0.1	1500	-	1635	3 × 1500	1.5
CL-GN0.3	1500	-	1615	3 × 1500	4.5
CL-GN0.5	1500	-	1601	3 × 1500	7.5
HL-R	-	1500	1015	3 × 1500	-
HL-GN0.1	-	1500	1015	3 × 1500	1.5
HL-GN0.3	-	1500	1015	3 × 1500	4.5
HL-GN0.5	-	1500	1015	3 × 1500	7.5

**Table 2:** A summary of the experimental tests carried out on hardened mortars.

Property	Symbol	Unit	* ECU (%)	Standard/Method
Bulk density	*ρ*_b_	kg∙m^−3^	1.4	EN 1015-10 [39]
Specific density	*ρ*_m_	kg∙m^−3^	1.2	He pycnometry
Open porosity	*ψ*	%	2.0	EN 1015-10/He pycnometry
Pore size diameter	*d*_p_	cm^3^∙g^−1^	-	Mercury intrusion porosimetry (MIP)
Average pore diameter	*d*_a_	µm	-	MIP
Pore size distribution	*PSD*	%	-	MIP
Flexural strength	*f*_f_	MPa	1.4	EN 1015-11 [40]
Compressive strength	*f*_c_	MPa	1.4	EN 1015-11 [40]
Young’s modulus	*E*_d_	GPa	2.3	EN 12504-4 [41]
Thermal conductivity	*λ*	W∙m^−1^∙K^−1^	-	Transient pulse technique
Volumetric heat capacity	*C*_V_	MJ∙m^−3^∙K^−1^	-	Transient pulse technique
Water absorption coefficient	*A*_w_	kg∙m^−2^∙s^−1/2^	2.3	EN 1015-18 [42]
24 h water absorption	*WA*_24_	kg∙m^−2^	2.3	EN 1015-18 [42]
Apparent moisture diffusivity	*κ*	m^2^∙s^−1^	2.3	Capillary water uptake [43]
Phase 1 drying rate	*D*_1_	kg·m^−2^·h^−1^	-	EN 16322 [44]
Phase 2 drying rate	*D*_2_	kg·m^−2^·h^−1/2^	-	EN 16322 [44]
Drying index	*DI*	-	-	EN 16322 [44]

* ECU—expanded combined uncertainty.

**Table 3:** Results of the XRF analysis of the binders and aggregate used (mass %).

Material	CL 90-S	HL 5	Silica Sand
CaO	95.32	57.86	0.01
Al_2_O_3_	3.06	11.63	3.24
SiO_2_	0.27	20.44	96.30
MgO	1.23	1.48	0.37
Fe_2_O_3_	0.05	1.77	0.04
SrO	0.04	0.05	-
NiO	0.03	-	-
Na_2_O	-	2.39	-
K_2_O	-	2.31	0.02
SO_3_	-	1.44	0.01
MnO	-	0.33	-
TiO_2_	-	0.26	0.01
ZrO	-	0.07	-
∑	100	100	100

**Table 4:** Basic physical characteristics of the binders and aggregate used.

Material	Loose Bulk Density (kg·m^−3^)	Specific Density (kg·m^−3^)	Blaine Specific Surface (m^2^·kg^−1^)
CL 90-S	446	2227	1652
HL 5	787	2624	357
Silica sand	1670	2647	-

**Table 5:** The basic properties of hardened mortars.

Mortar	Bulk Density *ρ*_b_ (kg·m^−3^)		Specific Density *ρ*_s_ (kg·m^−3^)		Porosity *ψ* (%)	
	28 Days	365 Days	28 days	365 Days	28 Days	365 Days
CL-R	1656 ± 23	1675 ± 23	2545 ± 31	2552± 31	34.9 ± 0.7	34.4 ± 0.7
CL-GN0.1	1647 ± 23	1661 ± 23	2545 ± 31	2557± 31	35.2 ± 0.7	35.0 ± 0.7
CL-GN0.3	1669 ± 23	1684 ± 23	2538 ± 35	2548± 31	34.2 ± 0.7	33.9 ± 0.7
CL-GN0.5	1641 ± 23	1659 ± 23	2536 ± 30	2547± 31	35.3 ± 0.7	34.9 ± 0.7
HL-R	1740 ± 24	1754 ± 24	2575 ± 31	2575± 31	32.4 ± 0.6	31.9 ± 0.6
HL-GN0.1	1913 ± 27	1935 ± 27	2572 ± 31	2597± 31	25.6 ± 0.5	25.5 ± 0.5
HL-GN0.3	1903 ± 27	1920 ± 27	2563 ± 31	2587± 31	25.8 ± 0.5	25.7 ± 0.5
HL-GN0.5	1871 ± 26	1891 ± 26	2531 ± 31	2582± 31	26.1 ± 0.5	25.9 ± 0.5

**Table 6:** Microstructural parameters obtained by mercury intrusion porosimetry (MIP).

Mortar	Total Pore Volume *d*_p_ (cm^3^·g^−1^)	Average Pore Diameter *d*_a_ (µm)
CL-R	0.193	0.217
CL-GN0.1	0.191	0.199
CL-GN0.3	0.184	0.189
CL-GN0.5	0.187	0.213
HL-R	0.171	0.068
HL-GN0.1	0.137	0.047
HL-GN0.3	0.138	0.060
HL-GN0.5	0.154	0.077

**Table 7:** Mechanical characteristics of studied mortars.

Mortar	Flexural Strength *f*_f_ (MPa)		Compressive Strength *f*_c_ (MPa)		Dynamic Modulus of Elasticity *E*_d_ (GPa)	
	28 Days	365 Days	28 Days	365 Days	28 Days	365 Days
CL-R	0.48 ± 0.01	0.89 ± 0.01	0.57 ± 0.01	1.86 ± 0.03	4.2 ± 0.1	4.5 ± 0.1
CL-GN0.1	0.70 ± 0.01	1.05 ± 0.01	0.75 ± 0.01	2.03 ± 0.03	4.8 ± 0.1	4.9 ± 0.1
CL-GN0.3	0.63 ± 0.01	1.03 ± 0.01	0.85 ± 0.01	2.13 ± 0.03	4.7 ± 0.1	4.9 ± 0.1
CL-GN0.5	0.63 ± 0.01	0.98 ± 0.01	0.82 ± 0.01	2.02 ± 0.03	4.6 ± 0.1	4.7 ± 0.1
HL-R	2.04 ± 0.03	2.33 ± 0.03	7.43 ± 0.10	10.20 ± 0.14	10.3 ± 0.3	10.7 ± 0.3
HL-GN0.1	2.14 ± 0.03	2.37 ± 0.03	10.98 ± 0.15	12.62 ± 0.18	12.7 ± 0.3	13.0 ± 0.3
HL-GN0.3	2.25 ± 0.03	2.41 ± 0.03	11.19 ± 0.15	13.25 ± 0.19	12.5 ± 0.3	13.0 ± 0.3
HL-GN0.5	2.13 ± 0.02	2.35 ± 0.02	9.80 ± 0.13	10.89 ± 0.15	12.1 ± 0.3	12.8 ± 0.3

**Table 8:** Thermal characteristics of 365-day mortars.

Mortar	Thermal Conductivity *λ* (W·m^−1^·K^−1^)	Volumetric Heat Capacity C_v_ × 10^6^ (J·m^−3^·K^−1^)
CL-R	1.157	1.106
CL-GN0.1	1.200	1.172
CL-GN0.3	1.227	1.205
CL-GN0.5	1.267	1.208
HL-R	1.106	1.063
HL-GN0.1	1.394	1.352
HL-GN0.3	1.409	1.366
HL-GN0.5	1.420	1.360

**Table 9:** The water absorption characteristics of 365-day cured mortars.

Mortar	Water Absorption Coefficient *A*_w_ × 10 (kg·m^−2^·s^−1/2^)	24 h Water Absorption *WA*_24_ (kg·m^−2^)
CL-R	0.32 ± 0.01	7.36 ± 0.17
CL-GN0.1	0.37 ± 0.01	8.74 ± 0.20
CL-GN0.3	0.34 ± 0.01	8.05 ± 0.19
CL-GN0.5	0.35 ± 0.01	9.12 ± 0.20
HL-R	0.08 ± 0.002	6.29 ± 0.11
HL-GN0.1	0.09 ± 0.002	6.54 ± 0.15
HL-GN0.3	0.09 ± 0.002	6.77 ± 0.16
HL-GN0.5	0.09 ± 0.002	6.57 ± 0.15

**Table 10:** The water absorption characteristics of the mortars cured for 365 days.

Mortar	Phase 1 Drying Rate *D*_1_ (kg·m^−2^·h^−1^)	Phase 2 Drying Rate *D*_2_ (kg·m^−2^·h^−1/2^)	Drying Index *DI* (-)
CL-R	0.36	1.57	0.14
CL-GN0.1	0.36	1.74	0.15
CL-GN0.3	0.38	1.78	0.15
CL-GN0.5	0.38	1.75	0.15
HL-R	0.26	0.88	0.20
HL-GN0.1	0.31	1.04	0.17
HL-GN0.3	0.35	1.18	0.17
HL-GN0.5	0.33	1.17	0.17

## Data Availability

The data presented in this study are available on request from the corresponding author due to privacy.
